# MRI Tracking of FePro Labeled Fresh and Cryopreserved Long Term *In Vitro* Expanded Human Cord Blood AC133+ Endothelial Progenitor Cells in Rat Glioma

**DOI:** 10.1371/journal.pone.0037577

**Published:** 2012-05-25

**Authors:** Branislava Janic, Kourosh Jafari-Khouzani, Abbas Babajani-Feremi, A. S. M. Iskander, Nadimpalli Ravi S. Varma, Meser M. Ali, Robert A. Knight, Ali S. Arbab

**Affiliations:** 1 Cellular and Molecular Imaging Laboratory, Department of Radiology, Henry Ford Hospital, Detroit, Michigan, United States of America; 2 Athinoula A. Martinos Center for Biomedical Imaging, Department of Radiology, Massachusetts General Hospital, Harvard Medical School, Boston, Massachusetts, United States of America; 3 Department of Neurology, Henry Ford Hospital, Detroit, Michigan, United States of America; National Institute of Health, United States of America

## Abstract

**Background:**

Endothelial progenitors cells (EPCs) are important for the development of cell therapies for various diseases. However, the major obstacles in developing such therapies are low quantities of EPCs that can be generated from the patient and the lack of adequate non-invasive imaging approach for *in vivo* monitoring of transplanted cells. The objective of this project was to determine the ability of cord blood (CB) AC133+ EPCs to differentiate, *in vitro* and *in vivo*, toward mature endothelial cells (ECs) after long term *in vitro* expansion and cryopreservation and to use magnetic resonance imaging (MRI) to assess the *in vivo* migratory potential of *ex vivo* expanded and cryopreserved CB AC133+ EPCs in an orthotopic glioma rat model.

**Materials, Methods and Results:**

The primary CB AC133+ EPC culture contained mainly EPCs and long term *in vitro* conditions facilitated the maintenance of these cells in a state of commitment toward endothelial lineage. At days 15–20 and 25–30 of the primary culture, the cells were labeled with FePro and cryopreserved for a few weeks. Cryopreserved cells were thawed and *in vitro* differentiated or IV administered to glioma bearing rats. Different groups of rats also received long-term cultured, magnetically labeled fresh EPCs and both groups of animals underwent MRI 7 days after IV administration of EPCs. Fluorescent microscopy showed that *in vitro* differentiation of EPCs was not affected by FePro labeling and cryopreservation. MRI analysis demonstrated that *in vivo* accumulation of previously cryopreserved transplanted cells resulted in significantly higher R2 and R2* values indicating a higher rate of migration and incorporation into tumor neovascularization of previously cryopreserved CB AC133+ EPCs to glioma sites, compared to non-cryopreserved cells.

**Conclusion:**

Magnetically labeled CB EPCs can be *in vitro* expanded and cryopreserved for future use as MRI probes for monitoring the migration and incorporation to the sites of neovascularization.

## Introduction

Neovascularization or new blood vessel formation is highly regulated process that plays crucial role in tissue- and organogenesis during embryonic development, and in tissue repair and regeneration in adulthood. The discovery of circulating endothelial cells (ECs) in the peripheral blood of patients affected by various vascular diseases implicated that endothelial progenitor cells (EPCs) may play an important role in postnatal vascularization [1]–[5]. Since then numerous studies tried to define the source as well as the phenotypic and functional characteristics of the putative EPC [6]–[8]. Currently, it is accepted that highly proliferative, immature EPC population expresses CD133+/CD34+/VEGFR2+ markers, and these cells give rise to more mature CD133−/CD34+/VEGFR2+ cells [9]. Through the process of further differentiation EPCs down-regulate the expression of CD34 and AC133 [10], [11], and continuous *in vitro* culturing increases the expression of mature endothelial cell (EC) markers [12]. Once differentiated into mature ECs, EPCs were shown to promote repair of damaged endothelium [13]–[16] and were implicated as critical in adult, postnatal endothelial repair and vasculogenesis that accompanies ischemic conditions such as myocardial ischemia and infarction, limb ischemia, wound healing, atherosclerosis and tumor neovascularization [6], [9]. The migration of progenitor cells to sites of ischemia and active neovascularization was demonstrated in different conditions such as limb muscle ischemia [17], cardiomyopathy and myocardial ischemia [18], [19], stroke [20], and as reported by our group, in mouse and rat breast cancer [21], melanoma and glioma models [22], [23]. Tissue ischemia and the hypoxic microenvironment associated with these pathologies [22], [24], [25] create a strong signal for mobilizing EPCs to hypoxic sites and a favorable environment that promotes the EPCs' neovascularization potential. These events strongly depend on the SDF-1-CXCR4 signaling pathway [17], [22], [24], [26], [27]. Expression of SDF-1 chemokine is driven by hypoxia and is significantly up-regulated in tumors and vascular ischemic conditions [18], [22]. The angiogenic potential of EPCs can be exhibited by their direct incorporation into newly formed blood vessels and/or by a paracrine effect, where non-incorporated EPCs secrete additional growth and angiogenic factors [6], [28]. Altogether, due to their capacity to proliferate, circulate and differentiate into mature ECs and demonstrated close association with vascular health, EPCs seem like excellent candidates for developing cellular therapies for conditions that depend on neovascularization mechanisms. Major impeding factors in developing EPC based therapies are limited quantities of cells that can be generated from a single patient as well as the lack of adequate non invasive imaging approaches for *in vivo* monitoring of transplanted cells. Hence, to effectively utilize cord blood clinically, generating sufficient numbers of therapeutically relevant cells through defined cell culture systems for *in vitro* expansion, cryopreservation and banking, as well as developing clinically compliant imaging system for monitoring the *in vivo* biodistribution of transplanted cells are critically important.

**Figure 1:**
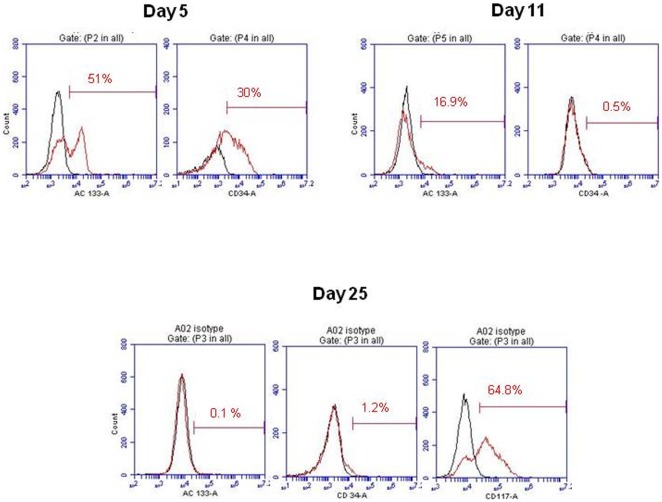
CB AC133+ EPCs-expression of cell surface markers during long term *in vitro* culture. The data depicts CD133 and CD34 protein expression levels in cells cultured for 5, 11 and 25 days and the levels of CD117 in cells cultured for 25 days (**A**). Flow cytometric histograms from one representative experiment are shown (n = 3). At least 10,000 live gated cells were analyzed for FITC, PE or PE-Cy5 expression. Isotype controls are shown as black histograms. Panel B shows cells induced to differentiate at day 25–30 of primary culture.

**Figure 2:**
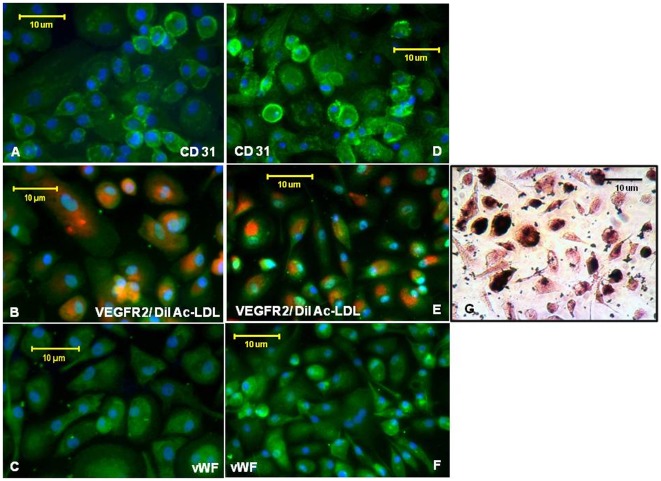
CB AC133+ EPCs expression of CD31, vWF and KDR and DiI-Ac-LDL uptake in differentiated progenitors – effect of FePro labeling and cryopreservation. Expression of CD31, VEGFR2 and vWF in differentiated cells that were prior to differentiating (at days 25–30 of the primary culture) labeled with FePro and cryopreserved for few weeks (D, E, F and G). Control cells were induced to differentiate at days 25–30 of the primary culture without previous FePro labeling and cryopreservation (A, B and C). Positive signals for CD31, VEGFR2 and vWF were visualized with a FITC conjugated secondary antibody (green). Nuclei were visualized with DAPI (blue). VEGFR2 positive (middle panels B and E) cells also exhibited the uptake of DiI-Ac-LDL (red). Representative photomicrographs (40×) of differentiated cells. Scale bar = 100 µm.

**Figure 3:**
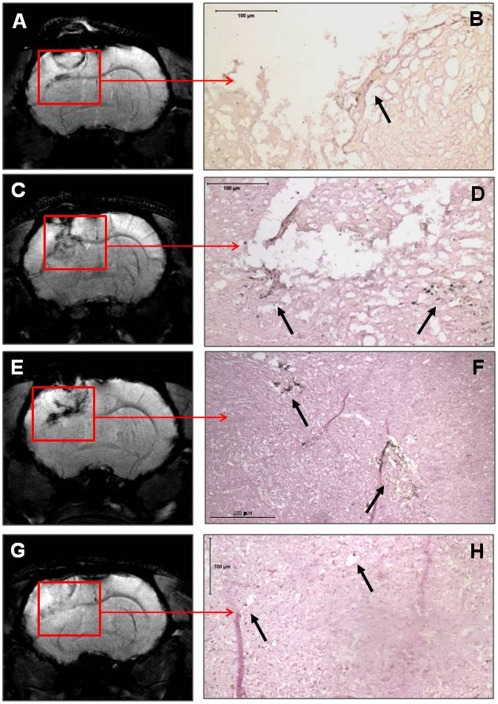
MRI detection of FePro labeled long-term cultured frozen and fresh CB AC133+ EPCs in glioma. At days 10–15 (A, B) and 25–30 (E, F) of the primary culture, cells were labeled with FePro and cryopreserved for few weeks. On the day of IV administration, the cells were thawed, incubated for 1–2 hours in stem cell media, washed and IV injected. A control group of rats received freshly prepared FePro labeled cells at 10–15 (C, D) and 25–30 (G, H) days of cultures. Seven days after cell administration multi-echo gradient-echo MRI were obtained using a 7 Tesla small animal MRI system. All animals receiving either frozen or fresh FePro labeled cells exhibited low signal intensity areas around tumors (arrows). Corresponding DAB enhanced Prussian blue stained sections showed iron positive cells at the tumor margins. Both frozen and fresh FePro labeled cells migrated and accumulated in tumor sites.

**Figure 4:**
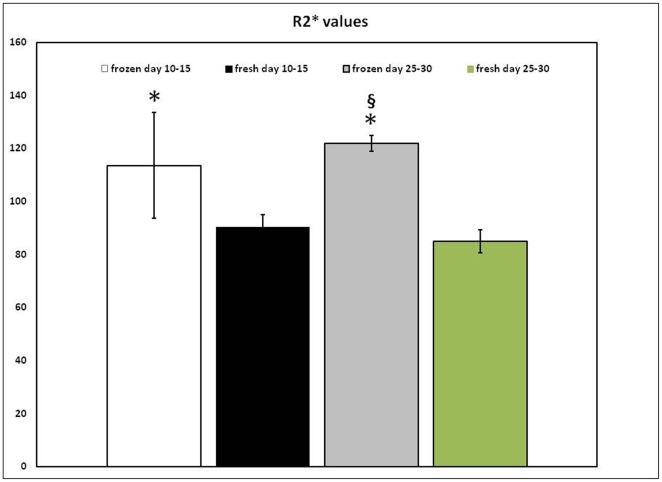
MRI relaxation parameters in tumors. Analyses of R2* values normalized to contralateral normal hemisphere (an indirect indicator of iron positive cell accumulation ) showed significantly higher (p<0.05) accumulation of iron positive cells in animals that received previously cryopreserved, FePro labeled CB AC133+EPCs that were *in vitro* expanded for 10–15 and 25–30 days. Bars: means ± SD. * p<0.05 frozen day 10–15 versus fresh day 25–30; **^§^** p<0.05 frozed day 25–30 versus fresh day 10–15 and fresh day 25–30.

**Figure 5:**
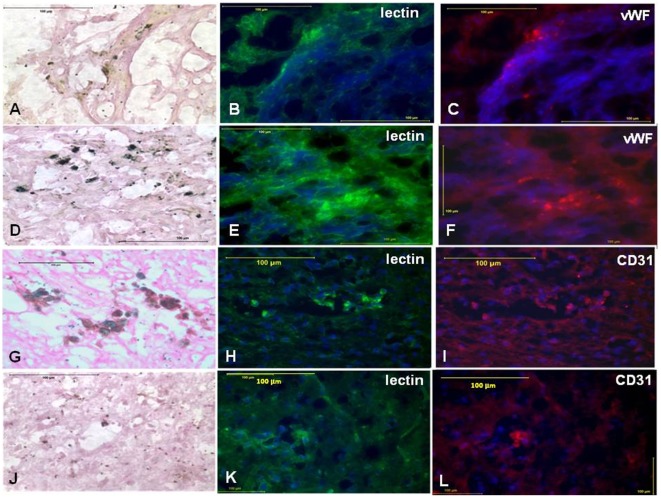
Effect of cryopreservation on *in vivo* angiogenic properties of CB AC133+ EPCs-immunohistology. At days 10–15 and 25–30 of the primary culture cells were labeled with FePro and cryopreserved for few weeks. Seven days after IV administration of thawed FePro labeled cells to glioma bearing rats, tissues were harvested and analyzed by DAB enhanced Prussian blue staining, FITC conjugated tomato lectin (green) and Rho conjugated antibodies that recognized vWF and CD31 expression. Panels A–C depict tissue sections of animals receiving frozen labeled cells that were cultured for 10–15 days. Control animals received 10–15 days cultured non-cryopreserved FePro labeled cells (D–F). Same experiments were done with cells expanded for 25–30 days. Tissue sections from the animals receiving frozen AC133+ are shown in panels G–I, while the section from control group receiving fresh cells are shown in J–L. Scale bar = 100 µm.

This work focuses on the isolation, long term expansion and cryopreservation of a single progenitor cell population derived from CB. Previously, we established the culture conditions for *in vitro* expansion of CB derived AC133+ progenitor cells that did not affect the ability of expanded cells to undergo endothelial differentiation and exhibit proangiogenic effects. The aim of this study was to further explore the effect of long term *ex vivo* expansion and cryopreservation on the ability CB AC133+ EPCs to migrate *in vivo* and incorporate in neovascularization sites in a rat glioma model. In addition, we used magnetic resonance imaging (MRI) in assessing these *in vivo* migratory and angiogenic properties of *ex vivo* expanded fresh and cryopreserved CB AC133+ EPCs.

## Materials and Methods

### Ethics Statement

The use of human cord blood in this study was approved by a Henry Ford Health System Institutional Review Board (IRB). Written informed consent was obtained and the consent process was maintained under IRB-approved security protocol IRB# 5805, using an IRB-approved consent form and the process of consent. Animal experiments were performed according to the protocol approved by our animal care and user committee at Henry Ford Health System, IACUC # 0963.

### Isolation and *In Vitro* Culture of CB AC133+ EPCs

Progenitor AC133+ cells were isolated from the cord blood obtained from volunteers under an Institutional Review Board (IRB) approved protocol. The cord blood mononuclear cell population was generated by Ficoll gradient centrifugation and was enriched for AC133+ cells by immunomagnetic positive selection using the MidiMACS system (Miltenyi Biotec, Auburn CA) according to the manufacturer's protocol. Upon isolation AC133+ cells were suspended in CellGro® SCGM media (CellGenix) supplemented with 40 ng/ml of stem cell factor (SCF), 40 ng/ml of FMS-like tyrosine kinase 3 (FLT3) and 10 ng/ml of thrombopoietin (TPO) (all from CellGenix). Cells were maintained as a suspension culture for 30 days with the cell concentration kept between 5×10^5^–1×10^6^ cells/ml. Upon determining the cell count, cells were split by adding freshly prepared media to adjust the concentration to 5×10^5^ cells/ml.

### Flow Cytometry

Cells expanded as suspension culture under the growth conditions described were harvested, washed in ice cold PBS and incubated for 30 min on ice, in dark, with the respective fluorescently labeled antibodies. Fluorescence activated flow cytometry was performed with a C6 Accuri flow cytometer (Accuri Inc., Ann Arbor, MI) and a minimum of 10,000 events were analyzed for each sample. Live cells used for the analysis were gated based on forward angle light scatter (FSC) and side angle light scatter (SSC) characteristics and further analyzed using the CFlow Plus Analysis Accuri software. Specific antibodies that were used in flow cytometric experiments to analyze the expression of cell surface markers were: mouse anti-human CD133 IgG1 (Miltenyi Biotec, Auburn CA), mouse anti-human CD34 IgG1 (BioLegend) and mouse anti-human CD117 IgG1 (BioLegend). All the antibodies were used in the concentrations suggested by the suppliers.

### *In Vitro* Differentiation of AC 133+ EPCs

At days 10–15 and 25–30 of primary long term *in vitro* expansion, AC133+ EPCs were either immediately induced to differentiate or labeled with FePro and cryopreserved for couple of weeks prior to *in vitro* differentiation. Differentiation was induced by suspending the cells in CellGro® SCGM media supplemented with 2% FBS and 2 ng/ml of Vascular Endothelial Growth Factor (VEGF) and plating in chamber slides coated with fibronectin at 1×10^5^/cm^2^. Cells were allowed to differentiate for 2 weeks. Every 2–3 days, old media was replenished with fresh media, and cells were monitored by inverted phase contrast microscopy to assess the morphological changes associated with differentiation. After 2 weeks of differentiation the cells were analyzed by fluorescence microscopy for the expression of EPC differentiation markers and DiI-Ac-LDL uptake.

### Immunocytochemistry

Differentiated CB AC133+ EPCs were analyzed by immunocytochemistry for the expression of endothelial cell specific markers. The following specific antibodies were used: mouse anti-human anti CD31 (DakoCytomation), rabbit anti-human anti CD309 (VEGFR2 or KDR) (Thermo Scientifics) and rabbit anti-human anti von Willebrand Factor (vWF) (DakoCytomation). Positive staining was detected using Rhodamine Red or FITC conjugated secondary antibodies (Jackson ImmunoResearch, Inc). Negative control samples included cells treated with secondary antibodies only. All the antibodies were used in the concentrations suggested by the suppliers. To visualize nuclei, cells were counterstained with DAPI nuclear stain. Cells were analyzed by fluorescent microscopy.

### Incorporation of DiI-Ac-LDL

Differentiated cells were incubated in the presence of 10 mg/ml of acetylated, DiI fluorescently labeled, low density lipoprotein (DiI-Ac-LDL) (Biomedical Technologies, Inc). After 4 h of incubation at 37°C, 5%CO_2_, the cells were washed with probe free-media, fixed in 3% paraformaldehyde and analyzed by fluorescent microscopy using rhodamine excitation/emission filters.

### Preparation of Ferumoxides-Protamine Sulfate (FePro) Complex and Labeling of CB AC133+ cells

At days 10–15 and 25–30 of primary culture, AC133+ cells were labeled according to our previously described method [29]. In brief, cells were suspended at the concentration of 4×10^6^ cell/ml in serum free RPMI and commercially available ferumoxides suspension (Fe) (Feridex IV; Bayer-Schering Pharma, Wayne, NJ, USA) was added to the cells at the final concentration of 100 µg/ml. Immediately after, preservative-free protamine sulfate (Pro) (American Pharmaceuticals Partners, Shaumburg, IL, USA) was added in the same manner to the final concentration of 3 µg/ml. Pro was supplied as 10 mg/ml of stock solution and was freshly diluted to a concentration of 1 mg/ml in distilled water at the time of use. Cells were plated in 24-well plate cell culture dish, 0.5 ml per well and incubated in the presence of FePro complexes for 15 minutes at 37°C, 5% CO_2_, after which complete growth media was added (0.5 ml per well) and the labeling procedure was further continued for 4 h at 37°C, 5% CO_2_. Upon labeling, cells were harvested, washed two times with 1× PBS and either cryopreserved or intravenously administered to glioma bearing nude rats. Cell labeling efficiency was determined by Prussian blue staining and by determining the intracellular iron concentration according to our published method [29]. Prussian blue staining was also employed for confirming the presence of intracellular iron in cells that were labeled with FePro and cryopreserved prior to differentiation.

### Cryopreservation of EPCs

Following *in vitro* culturing, cells were harvested, washed twice with PBS and resuspended at 10×10^6^cells/ml in freezing media that contained 5% DMSO, 10% human serum albumin (HSA), 5% of 10% hydroxyethyl starch (Pentastarch)(Braun Inc, USA) and 70% of serum free basal CellGro® SCGM media (CellGenix). Cells were placed in cryogenic vials and “dump-frozen” from room temperature to −85°C at a slow cooling rate of 1°C/min after which vials were kept at the constant temperature of −85°C for few weeks. Before IV administration, cells were thawed by standard fast thawing method in a water bath at 37°C [30]. Cells were then incubated in complete stem cell growth media for 1–2 h at 37°C, 5% CO_2_, washed, suspended in 2 ml of sterile PBS and induced to differentiate or IV administered to the animals.

### Human Glioma U251 Cells

Human glioma cells (U251, a generous gift from Dr. Steve Brown, Henry Ford Hospital) were grown in Dulbecco's modified eagles medium (DMEM) supplemented with 10% fetal bovine serum (FBS) in 5% CO_2_ at 37°C in a humidified incubator. For implantation into rat brain, the cells were harvested and resuspended in serum free media. A total of 4×10^5^ cells in 5 µl was implanted into the rat brain.

### Animal model

All animal experiments and housing conditions were approved by the Institutional Animal Care and Use Committee (IACUC) of Henry Ford Health System. At day 0, athymic nude rats, 6–8 weeks of age and 150–170 g of weight (Charles River Laboratory, Inc.) were anesthetized by intraperitoneal injection using ketamin/xylazine (100 mg/kg ketamine, 15 mg/kg xylazine) and placed on a small animal stereotactic device (Kopf, Cayunga, CA). The surgical zone was shaved and swabbed with betadine solution, eyes coated with Lacri-lube. After draping, a 1-cm incision was made 2 mm to the right of the midline, 1 mm retro-orbitally and the skull was exposed with cotton-tip applicators. A HP-4 dental drill bit was used with a micromanipulator to drill a hole 3 mm to the right and 1 mm anterior to the bregma, with care not to penetrate the dura. A #2701 10 µL Hamilton syringe with a #4 point, 26 s gauge-needle containing 5 µl of 4×10^5^ of U251 human glioma tumor cells was lowered to the depth of 3.5 mm, then raised to the depth of 2.5 mm. The U251 cells were injected stepwise at a rate of 0.5 µL/30 sec. Two to three minutes after completing the injection, the syringe was withdrawn in a stepwise manner and the surgical hole was sealed with bone wax. Finally, the skull was swabbed with betadine before suturing the incision.

### Administration of FePro labeled EPCs

Eleven days after U251 human glioma tumor cells implantation, the animals (n = 10 per group) received an intravenous injection of 10×10^6^ of either fresh or previously cryopreserved FePro labeled CB AC133+ EPCs that have been expanded for 10–15 or 25–30 days under *in vitro* conditions. For injection of previously cryopreserved cells, cells were thawed, incubated in complete stem cell growth media for 1–2 h at 37°C and 5% CO_2_. The cells were then washed, suspended in 2 ml of sterile PBS and injected. The control group of animals received intravenous injection of 2 ml of sterile PBS. On day 18 animals underwent *in vivo* MRI.

### *In vivo* MR imaging and analysis

#### Image acquisition

Rats were studied by MRI 18 days after U 251 tumor implantation (7 days after IV administration of EPCs). The animals were anesthetized with 2.0% isoflurane in oxygen carrier gas and secured to a customized cradle. Core temperature was maintained at 37.0°C. MRI was performed using 20-cm bore superconducting magnet 7T (Magnex Scientific, Abingdon, UK) interfaced to Bruker console (Billerica, MA, USA). After positioning using a triplanar FLASH sequence, T2- and T2*- weighted images were acquired. Spin echo T2-weighted images (T2WI) were obtained using a standard two-dimensional Fourier transformation (2DFT) multi-slice (21slices) multiecho (4 echoes) MRI sequence (TE = 15, 30, 45, and 60 msec, TR = 2000 msec, 32 mm FOV, 1 mm slice thickness, 256×256 matrix, and NEX = 2). The T2*-weighted images (T2*WI) were obtained using a standard multislice (21 slices) multi gradient-echo (4 echoes) MRI (TE = 11, 22, 33, and 44 msec, TR = 5000 msec, 32 mm FOV, 1 mm slice thickness, 256×256 matrix, and NEX = 2).

#### Image Analysis

R2 (1/T2) and R2* (1/T2*) maps were created from the T2WI and T2*WI image sets, respectively. The R2 and R2* maps were created with a least square fit on a pixel-by-pixel basis using an exponential model of the time series extracted from the multi-echo T2-weighted spin-echo and gradient-echo images, respectively using our custom made software Eigentool (http://www.radiologyresearch.org/eigentool.htm). Supplemental data show the representative multi-echo T2*WI images and signal intensity changes obtained from animals that received either cryopreserved or fresh magnetically labeled EPCs (**Figure S1**). The R2 and R2* values were determined by hand drawn irregular ROIs encircling the tumors for every section that contained tumor. The tumor area R2 and R2* values were normalized to the corresponding contra-lateral brain regions to produce a ratio of tumor to normal brain.

### Immunohistochemistry and Prussian blue staining

For histological analysis of brain tissue, animals were euthanized immediately after MRI imaging session. Animals were intravenously or intraperitonealy administered 150–200 mg/kg of Pentobarbital and then perfused with 100 mL of saline and 100 ml of 3% paraformaldehyde. The whole brain was collected and fixed in 4% paraformaldehyde and 3% sucrose. The fixed brain was then placed in 200–400 g coronal rat-brain matrix (Activational Systems Inc., Warren, MI) and cut into 1-mm blocks for processing and paraffin embedding. Some tissues were also processed as frozen sections. The embedded blocks were serially cut into 6–15 µm thick sections and analyzed by Prussian blue staining for the presence of FePro labeled administered CB AC133+ EPCs. Consecutive tissue sections were evaluated by standard immunofluorescence staining techniques for the expression of von Willebrand factor (vWF) and CD 31 using rabbit anti-human anti-vWF and mouse anti-human anti-CD31 antibodies (both from DAKOCytomation). Endothelial lining was also detected by FITC conjugated tomato lectin (Sigma). Prussian blue staining was performed according to our previously reported method [31].

### Statistical Analysis

Sample size was 10 animals per each group. Data are expressed as mean ± SD. Statistically significant difference was determined with one way or multi ANOVA analysis followed by Fisher's PLSD post-hoc test. A p-value of <0.05 was considered significant.

## Results

### Long term expanded CB AC133+ EPCs actively proliferate and differentiate into endothelia-like cells

To further explore the effect of long term *ex vivo* expansion, FePro labeling and cryopreservation on the ability of EPCs to differentiate *in vitro* towards ECs, CB AC133+ EPCs were maintained *in vitro* in suspension culture for 30 days. Cells were occasionally analyzed by flow cytometry to monitor expression of CD133, CD34 and CD117 markers. Upon isolation, majority of cells exhibited the expression of AC133 and CD 34 (>90%). As expected, and in accordance with our previous experience [32], over time cells down-regulated the expression of AC133 and CD34. At day 5 in culture, expression of AC133 and CD34 was detected on 51% and 30% of cells, respectively, while at day 11 only 16.9% of cells expressed AC 133 and 0.5% of cells expressed CD34 (**Figure 1**). In comparison, the expression of CD117 (c-kit) marker was still detected on more than 60% of cells at day 25, indicating the presence of actively proliferating progenitors. The endothelial differentiation potential of long term CB AC133+ culture was characterized at day 10–15 and 25–30 of primary culture and compared to the differentiation potential of thawed cells that were previously labeled with FePro and cryopreserved at day 10–15 and 25–30 of primary culture. Cells were differentiated 2 weeks, and analyzed for the expression of mature EC specific markers. In addition, the uptake of DiI-Ac-LDL as an assessment of differentiated ECs' functional integrity [33], [34] was also analyzed. Fluorescent microscopy revealed that the majority of cells exhibited expression of CD31, VEGFR2 and vWF when they were induced to differentiate at days 10–15 (data not shown) and 25–30 (**Figure 2A–C**) of primary culture. As shown in Figure 2D–E, expression of the same markers was not affected by previous FePro labeling and cryopreservation. Both non-cryopreserved and FePro labeled cryopreserved differentiated cells exhibited uptake of DiI-Ac-LDL that appeared as uniform, perinuclear red fluorescence. In addition, DAB enhanced Prussian blue staining of FePro labeled cryopreserved differentiated cells demonstrated intracellularly incorporated iron (**Figure 2G**). These results indicate that long term *in vitro* culturing, FePro labeling and cryopreservation did not impede the CB AC133+ cells' potential to differentiate towards ECs.

### MRI and histological detection of migration and accumulation of FePro labeled, long term cultured cryopreserved and fresh CB AC133+ EPCs in rat glioma model

In all animals, T2- and T2*-weighted images (T2WI and T2*WI, respectively) detected growing glioma tumors within the brains. In addition, MRI of all animals that received either frozen or fresh FePro labeled cells showed low signal intensity areas within and around the tumor tissue due to accumulation of the administered cells. These hypointense regions were more pronounced on T2*WI (**Figure 3**, panels A, C, E and G) and were detected in all animals receiving FePro labeled cells, regardless of the time of *in vitro* cells expansion (10–15 versus 20–25 days) or fresh vs. cryopreserved. The presence of administered FePro labeled cells was confirmed by DAB enhanced Prussian blue staining of the tissue sections corresponding to the areas exhibiting hypointense voxels and it demonstrated multiple, iron positive cells within the periphery and inside the tumors (**Figure 3**, panels B, D, F and H).On the other hand, similar low signal intensity indicating the presence of iron positive cells was not detected in animals that received IV injection of non-labeled EPCs (**Figure S2**). Image analysis demonstrated that, normalized R2 and R2* values obtained from animals that received FePro labeled CB AC133+ EPCs that were previously *in vitro* expanded for 25–30 days and cryopreserved for couple of weeks, were significantly higher (p<0.05) compared to that of animals that received non-cryopreserved FePro labeled cells (**Figure 4**). Significant differences were observed compared to both, fresh cells expanded for 10–15 and 25–30 days. On the other hand, R2 and R2* values obtained from animals that received FePro labeled CB AC133+ EPCs previously *in vitro* expanded for 10–15 days and cryopreserved for couple of weeks, were significantly higher (p<0.05) compared only to that of the animals that received non-cryopreserved FePro labeled cells *in vitro* expanded for 25–30 days (R2* data depicted in **Figure 3**). No significant differences in R2 and R2* values were detected when the same were compared to non-cryopreserved FePro labeled cells *in vitro* expanded for 10–15 days (p = 0.065). To further determine whether the administered cells associated and/or incorporated into the tumor neovascular structures, tissue sections were analyzed for the expression of neoangiogenic and endothelial differentiation markers by immunohistochemistry. For these purposes tissue sections that were consecutive to those that positively stained with DAB enhanced Prussian blue (showing the presence of administered FePro labeled cells) were analyzed. Additional staining with tomato lectin revealed overlapping localization of iron positive cells with tumor associated vasculature that was indicative of neovascular incorporation of administered cells (**Figure 5**). In addition, the same iron positive cells co-localized with the strong immunoreactivity detected by staining with anti-human vWF and anti-human CD31 antibodies, indicating *in vivo* differentiation of transplanted EPCs. Similar results were observed between animals that received cyropreserved FePro labeled cells expanded for both 10–15 and 25–30 days, and animals that received fresh (non-cryopreserved) FePro labeled cells expanded for both 10–15 and 25–30 days.

## Discussion

Endothelial progenitor cells (EPCs) have been introduced as one of the best candidates for developing cell based therapies for various conditions that involve vasculogenesis. Yet, to be able to realize such therapies, sufficient numbers of matching EPCs need to be generated, cryopreserved, banked and readily be available for treatment. This proved to be a challenging task due to very low numbers of EPCs *in viv*o, and according to previous reports these cells are exceptionally rare in bone marrow and peripheral blood (<0.05% and ≤0.01% of mononuclear cells, respectively) [35], [36]. In recent years however, human umbilical cord blood (CB) has been established as a source of various types of stem/progenitor cells [37]–[41] that can provide higher numbers of EPCs [35], [42]. Although therapeutic potential has been ascribed to EPCs derived from various sources, new evidence indicates that CB may provide distinct advantages over other EPCs' sources in terms of ontogeny [43], higher telomerase activity and associated proliferation potential [44], [45] and lower risk of graft versus host disease [46], [47]. In addition, CB is the largest source of stem cells available and the use of CB EPCs may be extremely advantageous in elderly and sick whose endogenous adult stem cell supply and bone marrow response may be inadequate, depleted and/or exhausted [48], [49]. In addition, cord blood is available without risk to mother or infant [50] and the non-invasive nature of CB collection and potential for easy and efficient characterization and banking, grant CB derived stem cells a unique therapeutic prospect [51]. Currently available data also indicate that CB AC133+ cells may be one of the best candidates for developing therapeutics for vascular ischemic diseases, in particular. Although there is no consensus on optimal numbers of cells needed to produce successful *in vivo* therapeutic effects, *in vitro* cell amplification, as well as long term cryopreservation appear to be necessary steps in achieving optimal graft conditions.

Here we used our previously established method to isolate AC133+ EPCs from CB, resulting in a cellular population of more than 90% purity [32]. Upon isolation, CB AC133+ EPCs were expanded under previously established optimal culture conditions [32] for up to 30 days. At different time points in culture, cell phenotype was characterized by flow cytometry. The expression of AC133 and CD34 cell surface markers was down regulated over time, with the complete loss of expression by day 25 of culture. The observed down-regulation indicated the presence of more mature endothelial progenitors and was in agreement with previously reported EPCs' phenotypic changes during *in vitro* culturing [32], [35], [42], [52]–[54]. At the same time, the majority of cells still expressed CD117 (c-kit) marker that indicates the presence of actively proliferating progenitors. In addition, during 30 days of *in vitro* culturing CB AC133+ EPCs maintained the ability to give rise to the functional progeny, i.e. mature endothelial cells as the majority of cells expressed CD31, VEGF receptor 2 (KDR) and vWF factor after 2 weeks of differentiation. These markers are considered as the expression hallmarks of endothelial cell type [35] and their expression was not affected by prior FePro labeling and cryopreservation. In addition, long term expanded CB AC133+ cultured under differentiation conditions, with or without previous FePro labeling and cryopreservation, also exhibited uptake of DiI-Ac-LDL. Altogether, these data demonstrated that FePro labeling and cryopreservation did not affect the expected changes associated with EC differentiation, *i.e.* properties necessary to carry out *in vivo* angiogenic effects of mature endothelial cells.

Next, we hypothesized that cryopreservation of long term expanded CB AC133+EPCs would not attenuate their *in vivo* angiogenic properties and that magnetic cell labeling coupled with MRI would be valuable for real time *in vivo* assessment of migration and neovascular incorporation of these cells. Clinical application of EPCs is considered to be most achievable in conditions that are characterized by tissue ischemia where the therapeutic approach may involve using these cells as a regenerative tool for treating human vascular diseases or as a delivery vehicle or target to restrict vascular growth in tumors [55]–[57]. The common attribute in both, vascular and tumor pathologies, is that tissue ischemia increases SDF-1 *in situ* expression, generating a strong migratory signal for circulating EPCs [24], [58]. The capacity to migrate in response to such stimuli is crucial for the EPCs angiogenic potential and involvement in neovascularization processes [19], [59], [60]. Our previous studies on glioma tumor animal models showed that both locally [22] and systemically [61] administered FePro labeled CB AC133+ cells migrated and incorporated into the tumor vasculatures and this *in vivo* CB AC133+ cells' migratory potential correlated with the increased expression of SDF-1 within the glioma tumor tissue [22]. In addition, the latest clinical studies demonstrate increased mobilization of circulating EPCs in malignant glioma patients that correlate with tumor angiogenic activity [62]. Therefore, we opted to use a rat glioma model to assess the effect of cryopreservation on the *in vivo* functional aspects of long term expanded CB AC133+ EPCs, by employing MRI. MRI as a noninvasive imaging technique has been shown efficient in *in vivo* monitoring of temporal and spatial migration of stem and other cells labeled with ferumoxides [31], [63]–[66]. Magnetic labeling with FePro has also been shown to not alter cell metabolism, proliferation, viability, and differentiation capacity [29], [31]. In the current study we administered CB AC133+ EPCs that had previously been *in vitro* expanded (10–15 and 25–30 days), FePro labeled and cryopreserved to glioma bearing rats. In both, group of animals that received either non-cryopreserved or cryopreserved cells, cell migration and tissue incorporation at day 18 of tumor development was detected on MRI as a hypointense region. This hypointensity was mainly at the periphery of the tumor and was due to the significant shortening of the T2 and T2* relaxation times by the iron oxide incorporated within the endosomes of FePro labeled, administered cells. The hypointense area observed on MRI correlated with the area where iron positive cells were detected by Prussian blue staining. In addition, iron positive cells colocalized with ongoing tumor angiogenesis and expressed human endothelial markers vWF and CD31. These findings indicated *in vivo* differentiation of iron labeled EPCs towards a more mature endothelial phenotype. Together, the patterns observed by MRI and histopathology demonstrated infiltration and incorporation of FePro labeled CB AC133+ EPCs into tumor neovascularization that was not attenuated by previous cryopreservation of the cells. Interestingly, MRI analysis revealed that the animals which received FePro labeled cells that were cryopreserved before IV administration exhibited significantly higher R2 and R2* values, that would suggest higher numbers of accumulated cells within the tumors. The actual mechanism responsible for this phenomenon is unknown, but may involve better *in vivo* survival or proliferation of previously cryopreserved cells. Previous reports on transplantation of stem/progenitor cells derived from various sources have not established a significant *in vivo* advantage when cryopreserved cells were used. However, it is interesting to note that earlier work exploring the effects of cryopreservation on transplanted human heart cells demonstrated that cryopreservation increased cell proliferation and reduced the immunogenicity of transplants [67]. Cord blood derived stem cell transplants have also been shown to exhibit a lower incidence of graft-versus –host disease with allogeneic grafts than transplants generated from bone marrow or peripheral blood, despite the HLA disparity [46], [47]. Nevertheless, if cryopreservation can be utilized to further reduce or eliminate graft-versus –host disease incidence, it would be an extremely significant step towards clinical use of CB generated EPC transplants. In general, cryopreservation process is a critical step in long term preservation of any type of stem cells; however this step may be even more critical when it comes to CB derived cells since the cells are harvested at the time of birth, with intent of use at much later time point. Currently, the majority of cryopreservation protocols for CB derived cells have largely been adapted from methods originally designed for bone marrow or peripheral blood hematopoietic cells (HSCs) and most commonly involve freezing of minimally separated nucleated or mononuclear cells without further, more specific cell separation [68]. While cryopreservation of HSCs for clinical use is routinely performed [69], cryogenic procedures for other progenitor cells are still being defined at the laboratory level. In this study we focused on cryopreservation of CB derived AC133+ EPCs. The ultimate goal in optimizing cryogenic conditions is to develop protocols with translational potential and in compliance with current Good Manufacturing Practices (cGMP). Therefore, the majority of studies focused on using xeno-free (i.e. FBS free) media with low concentration of permeable cryoprotectant (CPA), such as DMSO [70], [71]. In addition, large molecular weight, non-permeable CPAs such as hydroxyethyl starch also proved beneficial in reducing the cell injury due to water crystallization and intracellular ice formation [72]. The present study demonstrates a cryopreservation method that utilizes media containing xeno-free components with low concentrations of CPA reagents to produce favorable cryopreservation conditions that enabled previously frozen cells to preserve and *in vivo* exhibit proangiogenic properties such as migration and vascular incorporation. In addition, the cryopreservation method described does not require complex equipment or procedures and all the components could be cGMP compatible and clinically relevant.

In summary, the effects of cryopreservation on *in vitro* and *in vivo* angiogenic properties of long term expanded CB AC133+ cells were evaluated. The study demonstrated that under *in vitro* conditions, the differentiation potential of long term expanded CB AC133+EPCs was not affected by cryopreservation. On the other hand, *in vivo* accumulation of previously cryopreserved transplanted cells resulted in significantly higher R2 and R2* values indicating higher rate of migration and neovascular incorporation of these cells. In addition, we show that FePro labeling of EPCs and their *in vivo* tracking by MRI in an animal glioma model may be valuable tool for exploring the role of EPCs in vasculo- and angiogenic processes. However, to better understand the mechanisms behind the results presented here, further investigation is warranted.

## Supporting Information

Figure S1 Multi-echo T2*W images and signal intensity changes. Changes in signal intensity at different TE (upper and middle panels) in tumors that received either fresh (upper panel) or frozen (middle panel) EPCs. Changes in signal intensity were plotted against echo time (TE), which shows similarity between fresh (blue diamond) and frozen (red square) FePro labeled EPCs.
(TIF)

Figure S2 **MRI and Prussian blue staining do not detect non-labeled EPCs.** MRI of brain and DAB enhanced Prussian blue staining of brains sections from representative animals that received non-labeled (upper panel) and FePro labeled EPCs. The FePro labeled EPCs appeared as low signal intensity areas in the tumor regions of animals that received FePro labeled EPCs and were confirmed by the presence of Prussian blue positive cells Low signal intensity areas were not observed in animals that received non-labeled EPCs. To determine the similarity of signal intensity changes on T2*-weighted images at different echo time (TE), average signal intensity was measured from the whole tumors in representative animals that received either fresh or frozen FePro labeled EPCs. The measure mean value of signal intensity (Y axis) was plotted against TE (X axis). The T2* were estimated from a least square fit on a pixel-by-pixel basis using an exponential model of the time series extracted from the multi-echo gradient-echo images. Similarly T2 maps were also created (**Figure S1**). To determine whether non-labeled EPCs would generate low signal intensity on MR images, sets of animals received IV administration of FePro labeled and non-labeled EPCs after tumor implantation. Animals underwent MRI (FIESTA, fast imaging employed in steady-state acquisition) right before and 7 days after IV administration of EPCs. Following the last MRI, animals were euthanized and brains were collected for histochemistry analysis. **Figure S2** shows the MRI and DAB enhanced Prussian blue staining of brain sections. Low signal intensity areas were observed in animals that received labeled EPCs (white dotted circles), whereas the animals that received non-labeled EPCs did not show such areas. It should be noted that none of the tumors showed any low signal intensity areas before cell administration. Histochemistry analysis confirmed the presence of iron positive cells in animals that received labeled EPCs (brown cells in lower panel).
(TIF)
