# Supplementary figures and images for: MRI Tracking of FePro Labeled Fresh and Cryopreserved Long Term In Vitro Expanded Human Cord Blood AC133+ Endothelial Progenitor Cells in Rat Glioma
Source: PLoS One. 2012 May 25;7(5):e37577. doi: 10.1371/journal.pone.0037577 (PMC3360770; doi:10.1371/journal.pone.0037577)

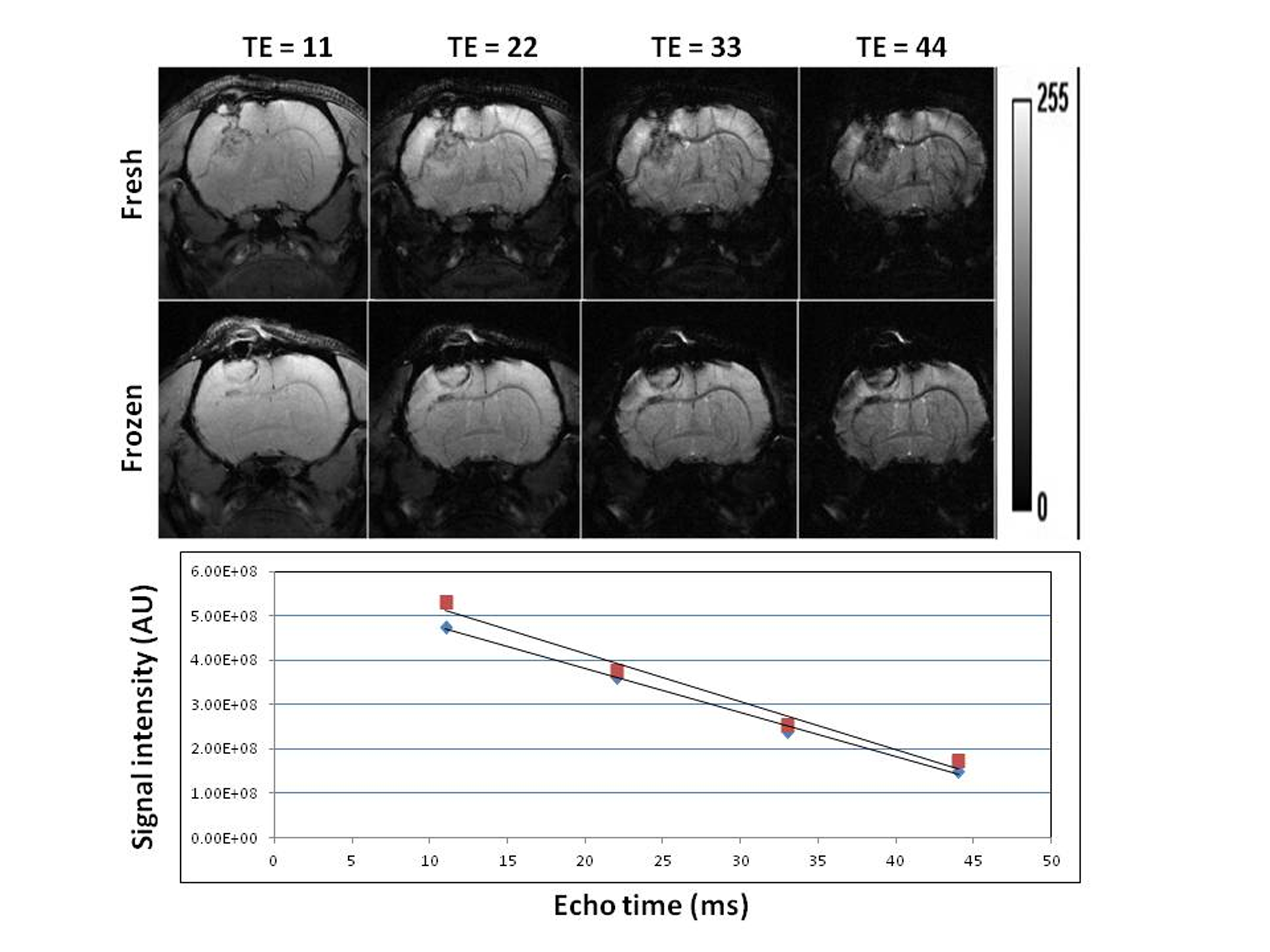

Supplement: Figure S1 — Multi-echo T2*W images and signal intensity changes. Changes in signal intensity at different TE (upper and middle panels) in tumors that received either fresh (upper panel) or frozen (middle panel) EPCs. Changes in signal intensity were plotted against echo time (TE), which shows similarity between fresh (blue diamond) and frozen (red square) FePro labeled EPCs. (TIF) [file pone.0037577.s001.tif]

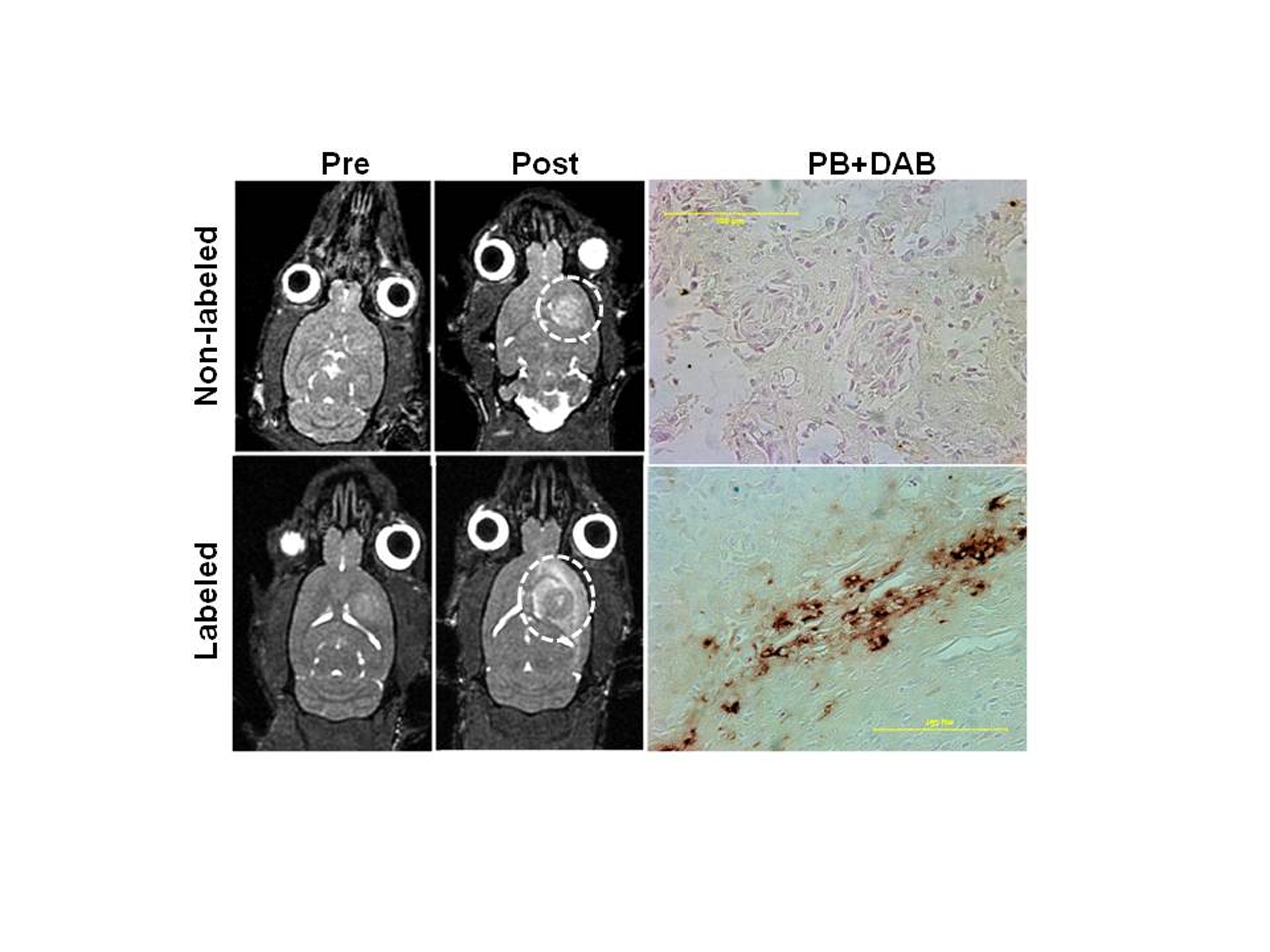

Supplement: Figure S2 — MRI and Prussian blue staining do not detect non-labeled EPCs. MRI of brain and DAB enhanced Prussian blue staining of brains sections from representative animals that received non-labeled (upper panel) and FePro labeled EPCs. The FePro labeled EPCs appeared as low signal intensity areas in the tumor regions of animals that received FePro labeled EPCs and were confirmed by the presence of Prussian blue positive cells Low signal intensity areas were not observed in animals that received non-labeled EPCs. To determine the similarity of signal intensity changes on T2*-weighted images at different echo time (TE), average signal intensity was measured from the whole tumors in representative animals that received either fresh or frozen FePro labeled EPCs. The measure mean value of signal intensity (Y axis) was plotted against TE (X axis). The T2* were estimated from a least square fit on a pixel-by-pixel basis using an exponential model of the time series extracted from the multi-echo gradient-echo images. Similarly T2 maps were also created (Figure S1). To determine whether non-labeled EPCs would generate low signal intensity on MR images, sets of animals received IV administration of FePro labeled and non-labeled EPCs after tumor implantation. Animals underwent MRI (FIESTA, fast imaging employed in steady-state acquisition) right before and 7 days after IV administration of EPCs. Following the last MRI, animals were euthanized and brains were collected for histochemistry analysis. Figure S2 shows the MRI and DAB enhanced Prussian blue staining of brain sections. Low signal intensity areas were observed in animals that received labeled EPCs (white dotted circles), whereas the animals that received non-labeled EPCs did not show such areas. It should be noted that none of the tumors showed any low signal intensity areas before cell administration. Histochemistry analysis confirmed the presence of iron positive cells in animals that received labeled EPCs (brown cells in low [file pone.0037577.s002.tif]
